# IL-6 Inhibits Starvation-induced Autophagy via the STAT3/Bcl-2 Signaling Pathway

**DOI:** 10.1038/srep15701

**Published:** 2015-11-09

**Authors:** Beibei Qin, Zhuo Zhou, Jianqin He, Chunlan Yan, Shiping Ding

**Affiliations:** 1 The National Education Base for Basic Medical Sciences, School of Medicine, Zhejiang University, Hangzhou 310058, Zhejiang Province, PR China

## Abstract

IL-6, a pleiotropic cytokine, has been investigated for its role in regulating autophagy. Yet, its mechanism of action remains unclear. Here, we show that IL-6 exerted anti-autophagic effects on U937 cells through the STAT3 signaling pathway *in vitro*. The addition of IL-6 to starved U937 cells significantly activated the phosphorylation level of STAT3 (p-STAT3) at Tyr705 and reduced the protein levels of microtubule-associated protein 1 light chain 3 of type II (LC3-II) and Beclin 1. By immunoblotting, we also observed a positive correlation between the p-STAT3 level and Bcl-2 level. Furthermore, treatment with a STAT3 inhibitor, LLL12, or overexpression of a mutant form, STAT3Y705F, reversed the inhibitory effect of IL-6 on autophagy. Knockdown of Beclin 1 or Atg14 by siRNA and over-expression of Beclin 1 indicated the involvement of class III PI3K complex in IL-6-mediated inhibition of autophagy. Taken together, these data indicate that IL-6 inhibits starvation-induced autophagy and that p-STAT3 mediates the signal transduction from IL-6 to downstream proteins including Bcl-2 and Beclin1.

Autophagy is a catabolic pathway conserved among eukaryotic cells^1^. This process is initiated by formation of the phagophore, which expands and fuses to form a vesicle called an autophagosome. Autophagosomes eventually fuse with lysosomes, which degrade their contents. Therefore, autophagy allows cells to rapidly eliminate long-lived proteins and destructive organelles for energy recycling^2^.

IL-6 is a pleiotropic cytokine that is mainly secreted by lymphocytes, monocytes and mononuclear macrophages after stimulation. IL-6 plays an important role in inflammation and participates in the pathogenesis of many diseases. Recently, it has been reported that IL-6 regulates the autophagic process through both inhibitory and stimulating effects on autophagy^3^,^4^,^5^. However, the molecular mechanisms of autophagic regulation by IL-6 remain unclear.

STAT3 is a member of the STAT protein family in humans^6^. In response to cytokines, STAT3 is phosphorylated by receptor-associated kinases, and it then forms homo- or hetero-dimers that translocate to the nucleus, acting as transcriptional activators. Recently, a succession of reports has indicated that STAT3 participates in the process of autophagy. These reports have proposed that a variety of cytokines can induce the phosphorylation of STAT3 (p-STAT3) at Ser727, promote its mitochondrial localization, and further activate autophagy^7^. Moreover, p-STAT3-mediated autophagy is completely inhibited by 3-MA and partially inhibited by knockdown of molecules involved in the autophagic processes^8^. However, opposing results have indicated that p-STAT3 has an inhibitory effect on autophagic flux. Fyn tyrosine kinase mediates STAT3 activation to further reduce the level of VPS34 protein, which inhibits skeletal muscle fiber-type-specific macroautophagy^9^. Furthermore, the inhibition of p-STAT3 induces autophagy^10^, and IL-6-induced STAT3 signaling has been associated with the inhibition of autophagic cell death, which attenuates arsenite-induced renal injury^3^. Additionally, it has been demonstrated that cytoplasmic non-phosphorylated STAT3, rather than p-STAT3, represses autophagy, as forced nuclear localization of STAT3 via the addition of a nuclear localization sequence failed to do so^11^. In brief, whether or not it is phosphorylated, STAT3 appears to be intimately involved in the autophagic process.

The IL-6 receptor (IL-6R) is a heterodimer, consisting of an 80-kD alpha subunit (IL-6Rα) and glycoprotein 130 (gp130). IL-6 interacts with the accessory transmembrane protein IL-6Rα and binds to the signal-transducing gp130 subunit with high affinity to form the IL-6/IL-6Rα/gp130 ternary complex, which activates downstream signal transduction pathways^12^. The main downstream signal transduction pathways of gp130 involve JAK/STAT, PI3K/Akt, and Ras/Erk signaling. These signals can be further amplified, resulting in changes in cellular activity. Among these signaling molecules, STAT3 is an important signaling protein downstream of the gp130 signaling pathway. The above-described research indicates that STAT3 also functions as a regulator of autophagy. Therefore, we inferred that STAT3 might play an important role in the process through which IL-6 regulates autophagy. To further identify the mechanism involved, we used U937 cells as a model to investigate the effect of IL-6 on autophagy.

## Results

### Interleukin-6 inhibited starvation-induced autophagy

**Figure 1:**
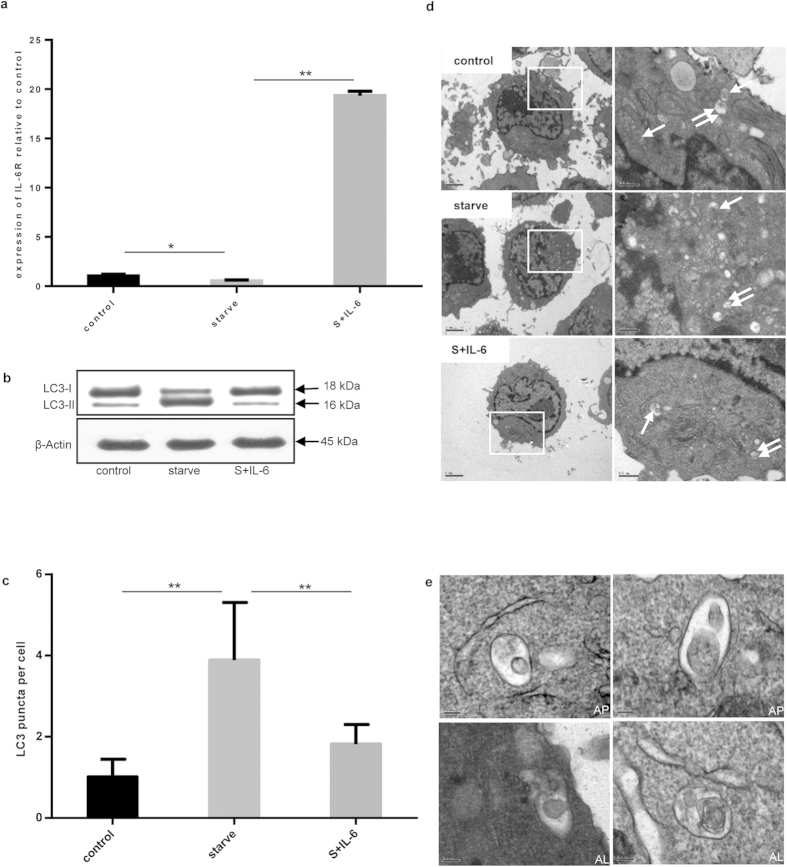
IL-6 inhibited starvation-induced autophagy. (**a**) The expression of IL-6R mRNA relative to β-actin was detected under starvation conditions (S) and incubation with or without IL-6 (30 ng/mL) for 2 h. Control versus starve, starve versus S+IL-6; *P < 0.05, **P < 0.01. (**b**) Immunoblot analysis of LC3 lipidation state. β-actin was used as the loading control. (**c**) The endogenous LC3 puncta per cell were quantified by immunofluorescence method. Control versus starve, starve versus S+IL-6; **P < 0.01. (**d**) Autophagosomes (single arrows) and autolysosomes (double arrows) were observed in starvation- or IL-6-treated cells were observed by TEM; Scale bars on the left side photos represent 2 μm and the right side ones represent 0.5 μm. (**e**) Detailed descriptions of autophagosomes (AP) and autolysosomes (AL); scale bars represent 0.1 μm.

First, the expression of IL-6R in U937 cells was ascertained by real-time PCR (Fig. 1a). Amino acid starvation reduced IL-6R expression to half that of the control cells. However, after IL-6 was added to cells in which autophagy had been induced by amino acid starvation, the expression of IL-6R increased.

The LC3-II/LC3-I ratio has been considered a hallmark of autophagy. The conversion of LC3-I into LC3-II was evident after amino acid starvation. When IL-6 was added to starved cells, the intensity of the LC3-I band increased while the LC3-II band decreased, indicating the inhibition of autophagy by IL-6 (Fig. 1b). We also detected the formation of endogenous LC3 puncta by immunofluorescence analysis. Specifically, amino acid starvation resulted in a significant increase in the number of endogenous LC3 puncta per cell, whereas the addition of IL-6 to starved U937 cells reduced the number of puncta, in keeping with the inhibition of autophagy (Fig. 1c). By transmission electron microscopy (TEM), we determined that several types of vesicles that were typically formed inside cells, such as autophagosomes and autolysosomes, were present (Fig. 1d,e).

**Figure 2:**
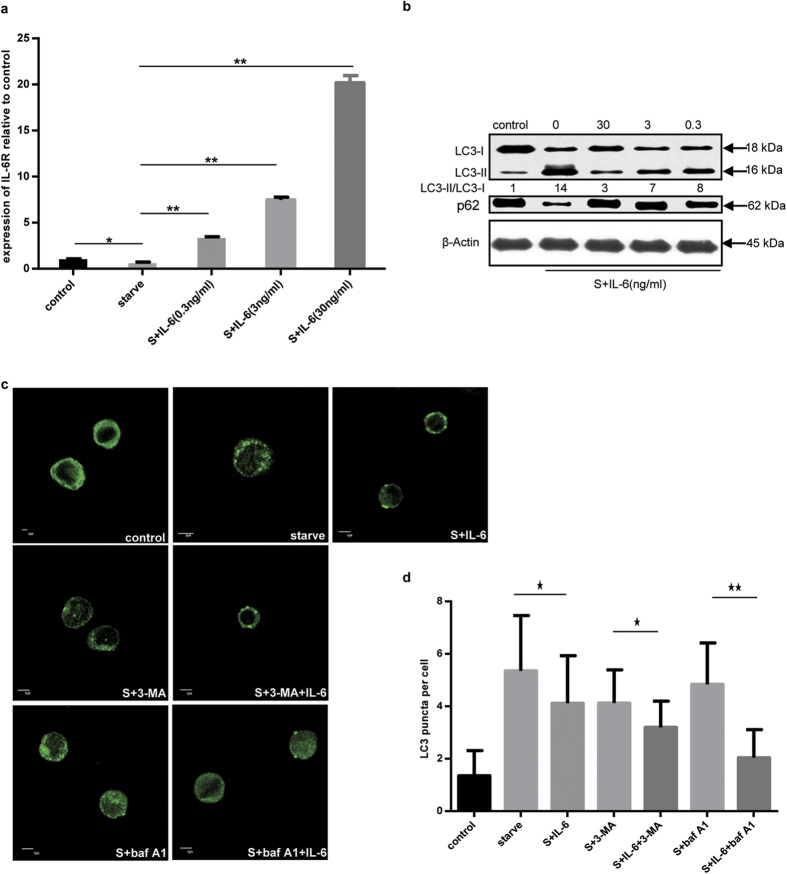
The relationship between IL-6 and autophagy. (**a**) The expression of IL-6R mRNA relative to β-actin was quantified by RT-PCR under starvation incubated with or without IL-6 at the indicated concentration for 2 h. (**b**) The LC3 lipidation state was assayed by immunoblotting under starvation conditions and incubation with or without IL-6 at the indicated concentration for 2 h. (**c**) The endogenous LC3 puncta were observed by immunofluorescence method under starvation conditions with or without IL-6 (30 ng/mL) or 3-MA (10 mM) or baf A1 (100 nM) for 2 h. (**d**) Endogenous LC3 puncta per cell were quantified. Scale bars represented 5 μm.

Titration of IL-6 in cells showed that IL-6R expression increased with the concentration of IL-6 in a dose-dependent manner (Fig. 2a). These results indicated that IL-6 might affect the functions of U937 cells via a specific receptor and its activity on cells might be regulated at the level of receptor-cytokine binding. A marked change in LC3-II intensity was shown, consistent with the inhibition of autophagy by IL-6. The expression of p62, another index for autophagic flux measurement, was also changed following the addition of IL-6 (Fig. 2b). In addition, we also found that the inhibitory effect of IL-6 on the starvation-induced autophagy functioned at the early stage of short-time exposure (about 2 h) (Supplementary Fig. 1).

To further demonstrate the effect of IL-6 on starvation-induced autophagy, 3-MA and baf A1 were added to the culture medium (Fig. 2c,d). Fewer endogenous LC3 puncta in amino acid-starved cells incubated with IL-6 were found in comparison to amino acid-starved cells. Furthermore, the combined effect of 3-MA and IL-6 reduced the number of endogenous LC3 puncta relative to that observed following 3-MA treatment. When baf A1 was added, an additional increase in endogenous LC3 puncta occurred under starvation conditions, while addition of IL-6 significantly reduced the number of fluorescent puncta in cells after 2 h of incubation. Together, these data further demonstrate that IL-6 inhibits starvation-induced autophagy.

### Inhibition of starvation-induced autophagy by IL-6 was STAT3-dependent

**Figure 3:**
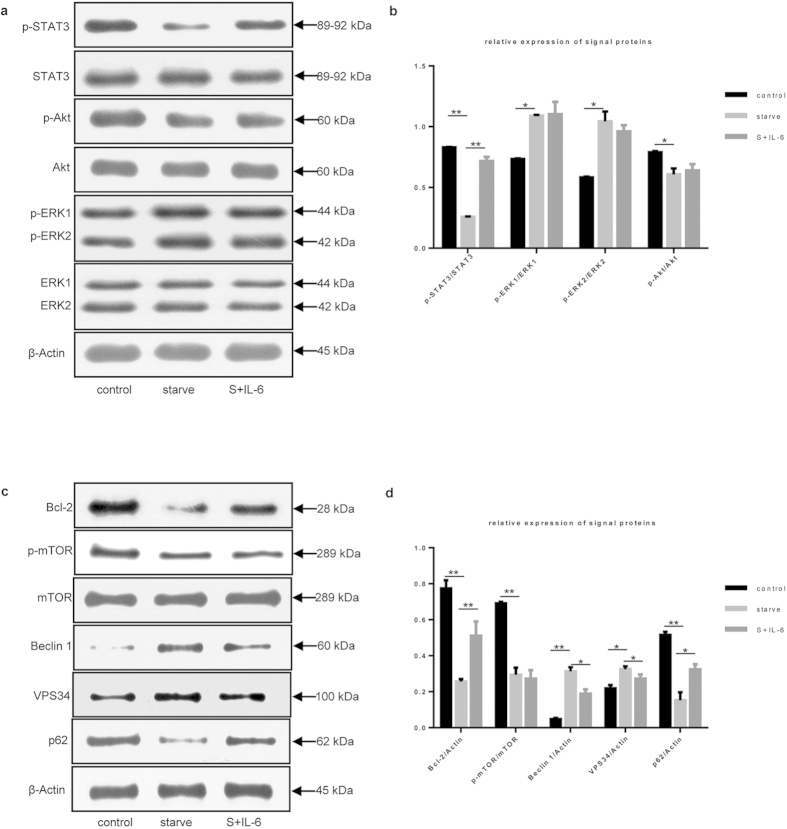
Inhibition of starvation-induced autophagy by IL-6 was STAT3- dependent. (**a**) STAT3, Akt and ERK1/2 were analyzed by immunoblotting under starvation conditions and incubation with or without IL-6 (30 ng/mL) for 2 h. β-actin was used as the loading control. (**b**) Relative expression levels of STAT3, Akt and ERK1/2 were shown in accordance with immunoblot analysis in Fig. 3a. Control versus starve, starve versus S+IL-6; *P < 0.05, **P < 0.01. (**c**) Immunoblot analysis of mTOR, Bcl-2, Beclin 1, VPS34 and p62 in cells treated with or without IL-6. (**d**) Relative expression levels of mTOR, Bcl-2, Beclin 1, VPS34 and p62 were shown in accordance with immunoblot analysis in Fig. 3c. Control versus starve, starve versus S+IL-6; *P < 0.05, **P < 0.01.

To determine the signaling pathway involved in the action of IL-6 on starvation-induced autophagy in U937 cells, we examined the activation of key downstream signal proteins including ATK, ERK1/2 and STAT3^13^,^14^,^15^,^16^,^17^. We found that starvation slightly changed the p-AKT, p-ERK1, and p-ERK2 levels in U937 cells (Fig. 3a,b); of note, the p-STAT3 level significantly decreased under the same conditions. The addition of IL-6 had little effect on the p-ERK1, p-ERK2, and p-AKT levels but markedly increased the p-STAT3 level relative to that observed following starvation. These data further suggest that STAT3 may be an important factor in the inhibition of starvation-induced autophagy by IL-6.

STAT3 has been reported to be a transcriptional activator of Bcl-2^18^,^19^, and its activation induces Bcl-2 expression^20^,^21^. Therefore, STAT3 is believed to positively regulate the expression of Bcl-2. We assessed the expression of Bcl-2 protein and found that the intensity change of Bcl-2 coincided with that of p-STAT3 (Fig. 3c), which further confirmed the relationship between these factors.

Furthermore, Bcl-2 has been recognized as an anti-autophagic effector protein as it interacts directly through its BH3 domain with Beclin 1, the key activator of autophagy^22^,^23^. This association of Bcl-2 with Beclin 1 blocks autophagy induction. Our results showed that the Bcl-2 level was reduced under starvation conditions and increased after the addition of IL-6, which was the opposite of the intensity changes observed for LC3-II under the same conditions. These data implied an anti-autophagic effect of Bcl-2.

Therefore, we inferred that Bcl-2 might mediate autophagy-related signal transduction from STAT3 to the class III PI3K complex containing Beclin 1 and VPS34. We next examined the expression of Beclin 1, VPS34 and p62. When the p-STAT3 level was reduced, the Beclin 1 and VPS34 expression levels were greatly increased under starvation conditions (Fig. 3c,d), whereas p62 expression was decreased. The addition of IL-6 to starved U937 cells reversed this effect, with an evident increase in p-STAT3 and decrease in VPS34 and Beclin 1; the p62 level was also increased under these conditions. These data indicate that Beclin 1 and VPS34 participate in the inhibition of starvation-induced autophagy mediated by IL-6.

Overall, these data support the hypothesis that IL-6 negatively regulates autophagy and that signaling via STAT3 activates Bcl-2, which in turn regulates Beclin 1 and VPS34 to control autophagy.

### Blockade of STAT3 signaling reversed IL-6 inhibition of starvation-induced autophagy

**Figure 4:**
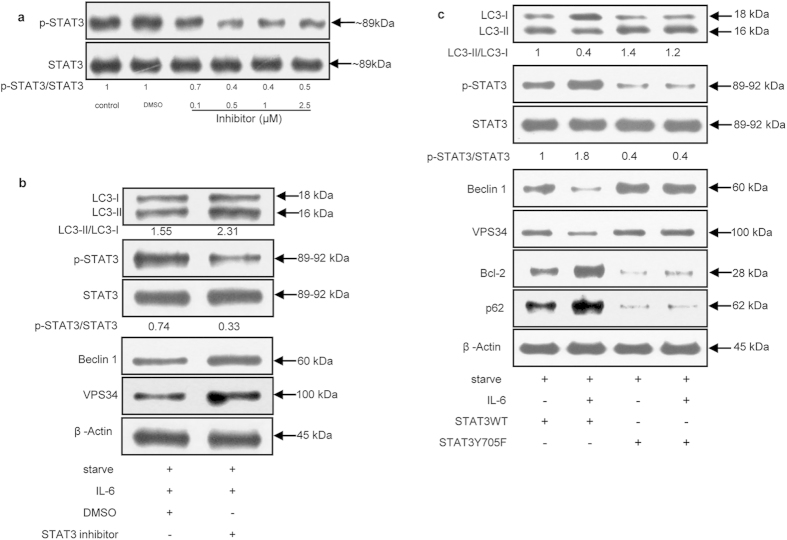
Blockade of STAT3 signaling reversed IL-6-mediated inhibition of starvation-induced autophagy. (**a**) Immunoblot analysis of STAT3 in U937 cells treated or not with LLL12 at the indicated concentration for 30 min or DMSO as a control before incubation in culture medium. (**b**) Immunoblot analysis of LC3, STAT3, Beclin 1 and VPS34 in U937 cells treated or not with LLL12 (0.5 μM) or DMSO for 30 min before incubation in culture medium. (**c**) Immunoblot analysis of STAT3, LC3, p62, Bcl-2, Beclin 1 and VPS34 in U937 cells transiently transfected with pcDNA3.1(+) STAT3WT or pcDNA3.1(+) STAT3Y705F for 48 h before incubation in culture medium.

To further confirm whether the STAT3-dependent pathway was involved in the ability of IL-6 to inhibit starvation-induced autophagy, the STAT3 inhibitor LLL12 was used in the following experiments. LLL12 was shown to be a more effective antagonist of p-STAT3 at Tyr705 than WP-1066^24^. After treatment with 0.5 μM LLL12, the p-STAT3/STAT3 ratio significantly declined by 60% compared with that observed for other concentrations of LLL12 (Fig. 4a).

Addition of 0.5 μM LLL12 enhanced the levels of Beclin 1 and VPS34. In addition, LLL12 treatment increased the LC3-II/LC3-I ratio (Fig. 4b), demonstrating its pro-autophagic role by inhibiting the activation of STAT3.

We also constructed a plasmid expressing wild-type STAT3 (STAT3WT) and dominant-negative STAT3 (STAT3Y705F) to genetically assess the role of STAT3 signaling in IL-6-mediated inhibition of starvation-induced autophagy (Fig. 4c). STAT3 activity was assessed in U937 cells transfected with STAT3WT or STAT3Y705F. When cells were transfected with a plasmid expressing STAT3WT, the addition of IL-6 further increased the p-STAT3 levels, and the autophagic flux was significantly weakened by the decrease in the LC3-II/LC3-I ratio. The Beclin 1 and VPS34 levels also changed accordingly. On the contrary, when cells were transfected with a plasmid expressing STAT3Y705F, the level of p-STAT3 decreased significantly and the autophagic flux was greatly enhanced. These data point to the importance of STAT3 in the process by which IL-6 inhibits autophagy.

In IL-6-treated cells, compared with wild-type STAT3, overexpression of STAT3Y705F not only significantly enhanced the autophagic flux and expression of Beclin 1 and VPS34 but also decreased the Bcl-2 levels. These findings confirm the positive effect of p-STAT3 on Bcl-2 expression. Taken together, these data provide evidence that p-STAT3 mediates the inhibition of starvation-induced autophagy by IL-6.

### Beclin 1 and Atg14 were involved in the inhibition of starvation-induced autophagy by IL-6

It has been reported that the induction of autophagy is triggered by the class III PI3K complex, mainly via p150, PI3 kinase, Beclin 1, and Atg 14 or UVRAG^25^,^26^. To further investigate whether these molecules mediate signal transduction in the IL-6/STAT3 pathway, we knocked down Beclin 1 and Atg14 respectively by 50% using siRNAs *in vitro*.

**Figure 5:**
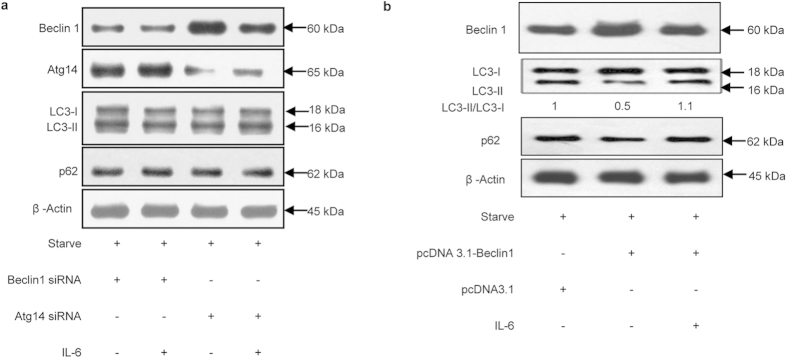
Beclin 1 and Atg14 were involved in the inhibition of starvation-induced autophagy by IL-6. (**a**) Immunoblot analysis of Beclin 1, Atg14, LC3 and p62 in U937 cells transiently transfected with or without Atg14 siRNA or Beclin 1 siRNA for 72 h before treatment. (**b**) Immunoblot analysis of Beclin 1, LC3 and p62 in U937 cells transiently transfected with pcDNA3.1-Beclin1 or pcDNA3.1 for 48 h before treatment.

Compared with Beclin 1 siRNA-treated samples, the addition of IL-6 had little effect on the LC3-II/LC3-I ratio and p62 level in starved U937 cells (Fig. 5a). Similar results were found when U937 cells were treated with Atg14 siRNA (Fig. 5a). These results indicated that knockdown of Beclin 1 or Atg14 abrogated the effects of IL-6 on starvation-induced autophagy, implying the involvement of these factors in IL-6-mediated inhibition of autophagy as components of the class III PI3K complex. Furthermore, compared with the empty vector control, cells transfected with Beclin 1 recombined plasmid highly expressed Beclin 1. Meanwhile, LC3II protein and the ratio of LC3II/LC3I increased while p62 protein decreased, indicating its enhancement of autophagy. The addition of IL-6 reduced protein levels of Beclin 1 and the ratio of LC3II/LC3I, demonstrating the involvement of Beclin 1 in IL-6 inhibiting autophagy (Fig. 5b). All these results demonstrated that Beclin 1 played an important role in the process of IL-6 on the autophagy.

In addition, Beclin 1 levels were reduced when cells were treated with IL-6 and Atg14 siRNA, suggesting that Beclin 1 might play a more important role in IL-6-mediated inhibition of autophagy than Atg14. Atg14 was also necessary for the process of autophagy.

**Figure 6:**
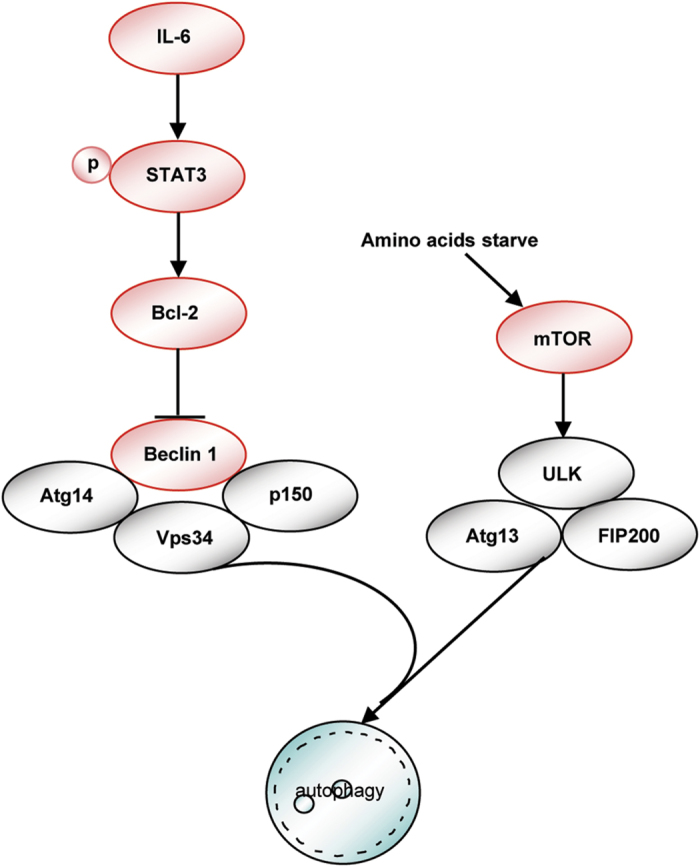
A schematic model showing the relationship between IL-6 and autophagy.

Together, these findings create a working model of the relationship between IL-6 and autophagy (Fig. 6).

## Discussion

Cytokines such as IFN-γ, IL-4, and IL-13 are associated with autophagic regulation, and recent reports have also demonstrated a role for IL-6 in this process^3^,^5^,^8^. This correlation between autophagy and IL-6 prompted us to pursue the mechanism by which IL-6 regulates autophagy.

Kimura *et al*. (2010) reported that the IL-6/STAT3 signaling pathway could inhibit autophagy but without any further research on downstream key signal molecules about the autophagic process. In this study, we provide evidence for the relationship between IL-6, p-STAT3, and autophagy. We show that IL-6 negatively regulates autophagy and that signaling via p-STAT3 activates Bcl-2 to regulate the expression of Beclin 1 and VPS34. In contrast, activation of the IL-6**/**p-STAT3 pathway was shown to be a positive regulator of autophagy in pancreatic cancer^5^. The different cell lines used in these studies may explain the discrepancy; the pancreatic cancer cells were derived from pancreatic parenchyma, whereas U937 cells were derived from the mononuclear phagocytic system. Additionally, differences in phosphorylation residues can cause cells to mount alternative responses to autophagy; in pancreatic cancer, secreted IL-6 induces p-STAT3 at Ser727, which was an important residue for its mitochondrial localization, whereas in this study, STAT3 activation was marked by its phosphorylation at Tyr705.

We have demonstrate that p-STAT3 participates in the regulation of autophagy as well. However, recent reports have shown that cytoplasmic STAT3, rather than p-STAT3, participates in autophagy^11^. Obviously, these results are conflicting and may be explained by the different cell lines and methods used. In addition, we present several important pieces of evidence showing that p-STAT3 is an important regulator of autophagy. First, p-STAT3 was significantly increased, whereas the LC3-II/LC3-I ratio was decreased with the addition of IL-6 relative to the starvation condition, implying the negative regulation of autophagy by p-STAT3. Second, a STAT3 inhibitor effectively decreased the level of p-STAT3 and further regulated the expression of autophagy-related proteins such as LC3-II, p62, Beclin 1, and VPS34. Third, when the mutant STAT3Y705F was transfected into starved U937 cells, it functioned as an autophagy inducer, while p-STAT3 worked as an autophagic inhibitor. However, further experimentation is still needed to support these findings.

Beclin 1 participates in autophagosome formation and mediates the localization of other autophagy proteins to the pre-autophagosomal membrane^27^. In this study, we found that Beclin 1 protein expression varied inversely with that of p-STAT3, which is in accordance with the reported regulation of Beclin 1 by p-STAT3^9^,^28^. Beclin 1 siRNA transfection further confirmed that STAT3 was an important upstream molecule in the inhibition of autophagy by IL-6. Yet, it remains unclear how STAT3 regulates Beclin 1 to further regulate the autophagic process. As reported, Bcl-2 is not only an anti-apoptotic protein but also an anti-autophagic protein via its inhibitory interaction with Beclin 1^22^,^29^. Consistent with this, the Bcl-2 level changed opposite to that of Beclin 1 in cells overexpressing STAT3Y705F. In addition, Bcl-2 has also been reported to be associated with STAT3, as activated STAT3 binds to the promoter of Bcl-2 and leads to its overexpression^30^. In this study, the intensity of Bcl-2 changed along with the phosphorylation state of STAT3. Thus, we propose that STAT3 mediates the inhibition of starvation-induced autophagy via the Bcl-2/Beclin 1 signal pathway in IL-6-treated U937 cells.

Our data indicate that IL-6 inhibits starvation-induced autophagy and activates p-STAT3 to regulate the effect of Bcl-2 on Beclin 1 and VPS34. This is the first report showing that IL-6 can regulate p-STAT3 to change the level of autophagy-related proteins and then further regulate the autophagy process.

## Material and Methods

### Reagents and antibodies

Recombinant human IL-6 was from Pepro Tech. LC3B; 3-MA and baf A1 were from Sigma Aldrich. Anti-ERK1/2, anti-phospho-ERK1/2, anti-Akt, and anti-phospho-Akt antibodies were from Bioworld Technology. Anti-STAT3, anti-phospho-mTOR, anti-mTOR, anti-p62, anti-Bcl-2, anti-Beclin 1, and anti-VPS34 antibodies were from Cell Signaling Technology (CST). Anti-phospho-STAT3 was from Wuhan Boster Biological Technology, Ltd. The STAT3 inhibitor LLL12 was from Merck Millipore. pcDNA3.1(+) (V790-20) was from Invitrogen.

### Cell culture and treatment

U937 cells (Shanghai Institutes for Biological Sciences) were maintained in RPMI-1640 (Invitrogen) supplemented with 10% heat-inactivated fetal bovine serum (Gibco), 100 U/mL penicillin, and 100 μg/mL streptomycin. Before use, the cells were differentiated with phorbol 12-myristate 13-acetate (100 nM) for 24–72 h. To induce autophagy, cells were incubated for 2 h in Earle’s balanced salt solution (EBSS) or treated with IL-6 at the indicated concentrations for 2 h. To analyze endogenous LC3 puncta with confocal microscopy, 3-MA (10 mM) or baf A1 (100 nM) was added to starved cells with or without IL-6 (30 ng/mL) for 2 h of incubation. For STAT3 phosphorylation, cells were either untreated or treated with LLL12 at the indicated concentrations or DMSO for 30 min before incubation in culture medium.

### Immunoblot analysis

After the treatment, cells were lysed in high-salt buffer, incubated on ice for 30 min, and microcentrifuged for 10 min at 4 °C to remove cell debris. Total protein concentration was measured with a kit (Bio-Rad Lab, Hercules, CA) in microtiter plates following the manufacturer’s protocol. A total of 70 μg protein was loaded and separated on a 15% SDS-polyacrylamide gel (BioRad). Then, proteins were electrotransferred to polyvinylidene difluoride membranes and incubated overnight with antibodies at 4 °C. Subsequently, the membranes were incubated with anti-rabbit IgG-peroxidase for 1 h at room temperature. Signals were detected using an enhanced chemiluminescence (ECL) kit.

### Transmission electron microscopy

Collected cells were fixed in 2.5% glutaraldehyde and kept overnight at 4 °C and then washed in 100 mmol/L phosphate buffer and post-fixed in 1% osmium tetroxide at 4 °C for 1 h. Then, the samples were dehydrated in a graded series of ethanols followed by propylene oxide, and embedded in resin. Ultrathin sections were stained with uranyl acetate and lead citrate and observed in a transmission electron microscope (Philips Tecnai10, Netherlands).

### Immunofluorescence analysis

U937 cells were incubated in culture medium or conditioned medium for the indicated times, collected, fixed with 4% paraformaldehyde for 15 min, permeabilized with 0.5% Triton X-100, and washed with phosphate buffer. After cell culture plates were blocked with 2% bovine serum albumin for 30 min, cells were incubated overnight with anti-LC3 antibody at 4 °C and subsequently washed with phosphate buffer. Anti-rabbit IgG-FITC antibody (Sigma Aldrich) was then added to the medium and incubated for 1 h at 37 °C. Images were collected on a laser-scanning confocal microscope (Olympus BX61W1-FV1000, Japan).

### Plasmid construction of pcDNA3.1 (+)-STAT3WT and pcDNA3.1 (+)-STAT3Y705F

A cDNA synthesis kit (TaKaRa) was used to reverse-transcribe mRNA into cDNA. Then, PCR was performed to amplify STAT3WT sequences with specific primers: forward 5′-CCGAAGCTTATGGCCCAATGGAATCAGC-3′ and reverse 5′-ATACTCGAGTCACATGGGGGAGGTAGCGCA-3′. STAT3 cDNA and pcDNA3.1(+) were digested by HindIII (TaKaRa) and XhoI (TaKaRa) enzymes, followed by linkage with T4 DNA ligase (TaKaRa). Genes were sequenced by Sangon Biology Technology Co. The primers for mutant STAT3Y705F were 5′-AGCGCTGCCCCATTCCTGAAGACCAAG-3′ (forward) and 5′-CTTGGTCTTCAGGAATGGGGCAGCGCT-3′ (reverse).

### Cell transfection

Following the manufacturer’s instructions, pcDNA3.1(+)-STAT3WT, pcDNA3.1 (+)-STAT3Y705F, pcDNA3.1-Beclin1 were transiently transfected into cells with Lipofectamine^TM^ 2000 (Invitrogen) for 48 h before incubation in culture medium.

Atg14 siRNA (CST), Beclin 1 siRNA (CST) or control siRNA (CST) were transfected into cells using siRNA transfection reagent (Roche) according to the manufacturer’s instructions. Then, cells were incubated for 72 h before treatments were administered.

### Real-time PCR

Cells were harvested after incubation in culture medium or conditioned medium for the indicated time. Total RNAs were isolated with Trizol. An RT reagent kit (Fermentas) was used to reverse-transcribe mRNA into cDNA. PCR was then performed using Realtime PCR Master Mix (Toyobo). The forward primer for Homo-IL-6R was 5′-GTACCACTGCCCACATTCCT-3′ and the reverse was 5′-TTCCACGTCTTCTTGAACCTC-3′. The forward primer for Homo-β actin was 5′-GCAGAAGGAGATCACTGCCCT-3′ and the reverse was 5′-GCTGATCCACATCTGCTGGAA-3′.

### Statistical analysis

Data were presented as means ± S.D. (approximately three independent experiments); Statistical comparisons were performed using Student’s t-test.

## Additional Information

**How to cite this article**: Qin, B. *et al*. IL-6 Inhibits Starvation-induced Autophagy via STAT3/Bcl-2 Signaling Pathway. *Sci. Rep*. **5**, 15701; doi: 10.1038/srep15701 (2015).

## Supplementary Material

Supplementary Information

## References

[b1] Klionsky D. J. & Emr S. D. Autophagy as a regulated pathway of cellular degradation. Science 290, 1717–1721 (2000). 11099404 10.1126/science.290.5497.1717 PMC2732363

[b2] Deretic V. Autophagy as an immune defense mechanism. Current Opinion in Immunology 18, 375–382 (2006). 16782319 10.1016/j.coi.2006.05.019

[b3] Kimura A. . The absence of interleukin-6 enhanced arsenite-induced renal injury by promoting autophagy of tubular epithelial cells with aberrant extracellular signal-regulated kinase activation. Am J Pathol 176, 40–50 (2010). 20008137 10.2353/ajpath.2010.090146 PMC2797868

[b4] Delk N. A. & Farach-Carson M. C. Interleukin-6: a bone marrow stromal cell paracrine signal that induces neuroendocrine differentiation and modulates autophagy in bone metastatic PCa cells. Autophagy 8, 650–663 (2012). 22441019 10.4161/auto.19226 PMC3405839

[b5] Kang R., Tang D., Lotze M. T. & Zeh H. J. 3rd. AGER/RAGE-mediated autophagy promotes pancreatic tumorigenesis and bioenergetics through the IL-6-pSTAT3 pathway. Autophagy 8, 989–991 (2012). 22722139 10.4161/auto.20258 PMC3427269

[b6] Zhong Z., Wen Z. & Darnell J. E. Jr. Stat3: a STAT family member activated by tyrosine phosphorylation in response to epidermal growth factor and interleukin-6. Science 264, 95–98 (1994). 8140422 10.1126/science.8140422

[b7] Kang R. . The expression of the receptor for advanced glycation endproducts (RAGE) is permissive for early pancreatic neoplasia. Proc Natl Acad Sci USA 109, 7031–7036 (2012). 22509024 10.1073/pnas.1113865109 PMC3345001

[b8] Yoon S. . STAT3 transcriptional factor activated by reactive oxygen species induces IL-6 in starvation-induced autophagy of cancer cells. Autophagy 6, 1125–1138 (2010). 20930550 10.4161/auto.6.8.13547

[b9] Yamada E. . Mouse Skeletal Muscle Fiber-Type-Specific Macroautophagy and Muscle Wasting Are Regulated by a Fyn/STAT3/Vps34 Signaling Pathway. Cell Rep 1, 557–569 (2012). 22745922 10.1016/j.celrep.2012.03.014 PMC3383827

[b10] Yokoyama T., Kondo Y. & Kondo S. Roles of mTOR and STAT3 in autophagy induced by telomere 3′ overhang-specific DNA oligonucleotides. Autophagy 3, 496–498 (2007). 17617738 10.4161/auto.4602

[b11] Shen S. . Cytoplasmic STAT3 represses autophagy by inhibiting PKR activity. Mol Cell 48, 667–680 (2012). 23084476 10.1016/j.molcel.2012.09.013

[b12] Kishimoto T. Interleukin-6: from basic science to medicine--40 years in immunology. Annu Rev Immunol 23, 1–21 (2005). 15771564 10.1146/annurev.immunol.23.021704.115806

[b13] Wullschleger S., Loewith R. & Hall M. N. TOR signaling in growth and metabolism. Cell 124, 471–484 (2006). 16469695 10.1016/j.cell.2006.01.016

[b14] Arico S. . The tumor suppressor PTEN positively regulates macroautophagy by inhibiting the phosphatidylinositol 3-kinase/protein kinase B pathway. J Biol Chem 276, 35243–35246 (2001). 11477064 10.1074/jbc.C100319200

[b15] He C. & Klionsky D. J. Regulation mechanisms and signaling pathways of autophagy. Annu Rev Genet 43, 67–93 (2009). 19653858 10.1146/annurev-genet-102808-114910 PMC2831538

[b16] Ogier-Denis E., Pattingre S., El Benna J. & Codogno P. Erk1/2-dependent phosphorylation of G alpha-interacting protein stimulates its GTPase accelerating activity and autophagy in human colon cancer cells. J Biol Chem 275, 39090–39095 (2000). 10993892 10.1074/jbc.M006198200

[b17] Pattingre S., Bauvy C. & Codogno P. Amino acids interfere with the ERK1/2-dependent control of macroautophagy by controlling the activation of Raf-1 in human colon cancer HT-29 cells. J Biol Chem 278, 16667–16674 (2003). 12609989 10.1074/jbc.M210998200

[b18] Perillo B., Sasso A., Abbondanza C. & Palumbo G. 17beta-estradiol inhibits apoptosis in MCF-7 cells, inducing bcl-2 expression via two estrogen-responsive elements present in the coding sequence. Mol Cell Biol 20, 2890–2901 (2000). 10733592 10.1128/mcb.20.8.2890-2901.2000 PMC85519

[b19] Alas S. & Bonavida B. Rituximab inactivates signal transducer and activation of transcription 3 (STAT3) activity in B-non-Hodgkin’s lymphoma through inhibition of the interleukin 10 autocrine/paracrine loop and results in down-regulation of Bcl-2 and sensitization to cytotoxic drugs. Cancer Res 61, 5137–5144 (2001). 11431352

[b20] Real P. J. . Resistance to chemotherapy via Stat3-dependent 10overexpression of Bcl-2 in metastatic breast cancer cells. Oncogene 21, 7611–7618 (2002). 12400004 10.1038/sj.onc.1206004

[b21] Sepúlveda P., Encabo A., Carbonell-Uberos F. & Miñana M. D. BCL-2 expression is mainly regulated by JAK/STAT3 pathway in human CD34+ hematopoietic cells. Cell Death Differ 14, 378–380 (2007). 16841088 10.1038/sj.cdd.4402007

[b22] Pattingre S. . Bcl-2 antiapoptotic proteins inhibit Beclin 1-dependent autophagy. Cell 122, 927–939 (2005). 16179260 10.1016/j.cell.2005.07.002

[b23] Maiuri M. C., Zalckvar E., Kimchi A. & Kroemer G. Self-eating and self-killing: crosstalk between autophagy and apoptosis. Nat Rev Mol Cell Biol 8, 741–752 (2007). 17717517 10.1038/nrm2239

[b24] Iwamaru A. . A novel inhibitor of the STAT3 pathway induces apoptosis in malignant glioma cells both *in vitro* and *in vivo*. Oncogene 26, 2435–2444 (2007). 17043651 10.1038/sj.onc.1210031

[b25] Liang C. . Autophagic and tumour suppressor activity of a novel Beclin1-binding protein UVRAG. Nat Cell Biol 8, 688–699 (2006). 16799551 10.1038/ncb1426

[b26] Itakura E. & Mizushima N. Atg14 and UVRAG: mutually exclusive subunits of mammalian Beclin 1-PI3K complexes. Autophagy 5, 534–536 (2009). 19223761 10.4161/auto.5.4.8062

[b27] Kihara A., Kabeya Y., Ohsumi Y. & Yoshimori T. Beclin-phosphatidylinositol 3-kinase complex functions at the trans-Golgi network, EMBO Rep 2, 330–335 (2001). 11306555 10.1093/embo-reports/kve061 PMC1083858

[b28] Lipinski M. M. . A genome-wide siRNA screen reveals multiple mTORC1 independent signaling pathways regulating autophagy under normal nutritional conditions. Dev Cell 18, 1041–1052 (2010). 20627085 10.1016/j.devcel.2010.05.005 PMC2935848

[b29] Pattingre S. & Levine B. Bcl-2 inhibition of autophagy: a new route to cancer? Cancer Res 66, 2885–2888 (2006). 16540632 10.1158/0008-5472.CAN-05-4412

[b30] Stephanou A., Brar B. K., Knight R. A. & Latchman D. S. Opposing actions of STAT-1 and STAT-3 on the Bcl-2 and Bcl-x promoters. Cell Death Differ 7, 329–330 (2000). 10866494 10.1038/sj.cdd.4400656

