# Supplementary material for: IL-6 Inhibits Starvation-induced Autophagy via the STAT3/Bcl-2 Signaling Pathway
Source: Sci Rep. 2015 Nov 9;5:15701. doi: 10.1038/srep15701 (PMC4637890; doi:10.1038/srep15701)
Supplement: Supplementary Information [file srep15701-s1.pdf]

# Supplemantary Information

## IL-6 Inhibits Starvation-induced Autophagy via the STAT3/Bcl-2 Signaling Pathway

Beibei Qin[1], Zhuo Zhou[1], Jianqin He[1], Chunlan Yan[1], Shiping Ding[1]*

The National Education Base for Basic Medical Sciences, School of Medicine, Zhejiang University, Hangzhou 310058, Zhejiang Province, PR China.

*Corresponding author: dingshiping@zju.edu.cn

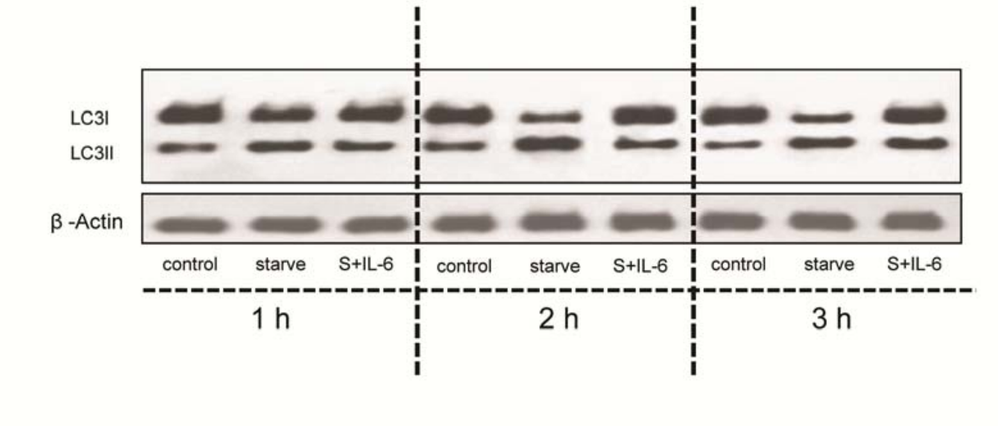


**Supplementary Fig.1** U937 cell was under starvation conditions and incubation with or without IL-6 (30 ng/mL) at the indicated concentration for 1 h, 2 h, 3 h. Western blotting was performed to determine the levels of LC3 lipidation state. β-actin was used as the loading control.
